# The impact of borderline personality disorder and sub-threshold borderline personality disorder on the course of self-reported and clinician-rated depression in self-harming adolescents

**DOI:** 10.1186/s40479-017-0073-5

**Published:** 2017-10-31

**Authors:** Ruth-Kari Ramleth, Berit Groholt, Lien M. Diep, Fredrik A. Walby, Lars Mehlum

**Affiliations:** National Centre for Suicide Research and Prevention, Institute of Clinical Medicine, University of Oslo, Sognsvannsveien 21, Bygg 12, 0372 Oslo, Norway

**Keywords:** Self-harm, Adolescents, Depression, Depressive symptoms, Borderline personality disorder, Borderline personality disorder traits, Dialectical behaviour therapy, Dialectical behaviour therapy adapted for adolescents, Sub-threshold BPD

## Abstract

**Background:**

Studies on adults suggest that the presence of comorbid depression and Borderline Personality Disorder (BPD) is associated with an elevated risk of self-harming behaviours and that self-harming behaviours, when present, will have higher severity. This comorbidity, furthermore, complicates clinical assessments, which may be an obstacle to early identification and proper intervention. Adolescents who self-harm frequently report high levels of depressive symptoms, but this is often not reflected in the clinicians’ assessment. BPD is still a controversial diagnosis in young people, and less is known about the clinical significance of comorbid BPD in adolescent populations.

The purpose of the present study was to examine the impact of BPD on the assessment and course of self-reported and clinician-rated depression in self-harming adolescents before and after a treatment period of 19 weeks. We hypothesized that, compared to adolescents without BPD, adolescents with BPD would self-report higher levels of depression at baseline, and that they would have less reduction in depressive symptoms.

**Methods:**

A total of 39 adolescents with depressive disorders and BPD-traits participating in a randomised controlled trial on treatment of self-harm with Dialectical Behaviour Therapy adapted for Adolescents or enhanced usual care were included. Adolescents with full-syndrome BPD (*n* = 10) were compared with adolescents with sub-threshold BPD (*n* = 29) with respect to their self-reported and clinician-rated depressive symptoms, suicidal ideation and global level of functioning at baseline, and after 19 weeks of treatment (end of trial period).

**Results:**

At baseline, adolescents with full-syndrome BPD self-reported significantly higher levels of depressive symptoms and suicidal ideation compared to adolescents with sub-threshold BPD, whereas the two groups were rated as equally depressed by the clinicians. At trial completion, all participants had a significant reduction in suicidal ideation, however, adolescents with BPD had a poorer treatment outcome in terms of significantly higher levels of clinician-rated and self-reported depressive symptoms and significantly lower levels of global functioning. At baseline as well as at trial completion, self-reported and clinician-rated levels of depressive symptoms were not significantly correlated in adolescents with BPD. In a multiple linear regression analysis, a diagnosis of BPD and a high baseline level of clinician-rated depressive symptoms predicted higher levels of depressive symptoms at trial completion, whereas receiving Dialectical Behaviour Therapy predicted lower levels of depressive symptoms.

**Conclusion:**

Our findings suggest that a diagnosis of BPD may have a strong impact on the assessment and course of depressive symptoms in self-harming adolescents. Although rated as equally depressed, adolescents with BPD self-reported significantly higher levels of depressive symptoms and suicidal ideation at baseline, and showed a poorer outcome in terms of higher levels of depressive symptoms and lower levels of global functioning at trial completion compared to adolescents with sub-threshold BPD. Our findings suggest that receiving Dialectical Behaviour Therapy could lead to a greater reduction in depressive symptoms, although firm conclusions cannot be drawn given the limited sample size.

Clinicians should be aware of the possibility of underestimating the severity of depression in the context of emotional and behavioral dysregulation. Providing BPD specific treatments seems to be important to achieve sufficient treatment response with regard to depressive symptoms in adolescents with BPD-traits.

**Trial registration:**

Treatment for Adolescents With Deliberate Self Harm; NCT00675129, registered May 2008.

## Background

The majority of adolescents reporting self-harming behaviours have engaged in these behaviours only once or a few times [1]. For some adolescents, however, self-harm has evolved into a maladaptive behavioural pattern with significant functional impairment and high risks of severe physical injury or death and a strong need for psychiatric and medical treatment [2]. Both community based [3] and clinical [1, 4] studies on adolescents have shown a strong association between self-harm and psychiatric disorders, especially with depressive disorders and Borderline Personality Disorder (BPD) [4, 5]. Studies on adults have shown that the two conditions frequently co-occur, and it is suggested that this comorbidity is associated with more frequent and more lethal self-harming behaviours [6, 7] and a worse prognosis [8, 9]. Studies suggest that among young people there is, similarly, a high prevalence of co-occurring depression and BPD [10, 11]. Assessment of psychiatric disorders in children and adolescents may, however, be challenging since mental disorders may manifest themselves differently, and symptoms may be more fluctuating and obscure in these age-groups. There is now general agreement that personality disorders have their origin in childhood and adolescence [12], and several studies have demonstrated that BPD can be reliably diagnosed in adolescent samples [13], with a diagnostic stability [14, 15], severity and comorbidity profile [16] comparable to adult samples. Nevertheless, many clinicians are still reluctant to diagnose BPD in adolescence [17, 18]. There is a need for more knowledge about the clinical significance of comorbid BPD in adolescent populations, since early identification and treatment will likely reduce long-term impairment and mortality [19].

Rating scales completed by patients and/or clinicians are often used to evaluate the severity of depression, to guide treatment choices, and to monitor changes during and following treatment. Although there is no gold-standard in the assessment of severity of depression, traditionally treatment choices have been guided by clinicians’ assessments, and clinical trials have traditionally relied on clinician-rated instruments to study treatment efficacy. However, clinical studies have shown that there is only a moderate correlation between patients’ and clinicians’ measurement of the level of depressive symptoms [20]. Significant discrepancies between self-reported and clinician-rated versions of the same scale suggest that factors other than differences in scale content play a role [21]. Detecting such discrepancies may add valuable clinical information, for instance predicting challenges building a therapeutic alliance [22, 23]. Studies on adults with BPD have shown that these patients score on average higher on self-report measures of depression than on clinician-rated measures [24–26]. Less is known about such discrepancies in adolescents with BPD.

Using a sub-sample of adolescents with a depressive disorder from a randomised trial on the efficacy of Dialectical Behaviour Therapy in adolescents, the purpose of the present study was to examine to what extent a comorbid BPD will be associated with more discrepancies between self-reported and clinician-rated levels of depression, and with the course of depressive symptoms during treatment. Depression was evaluated by comparing self-reported and clinician-rated levels of depression in self-harming adolescents before and after a treatment period of 19 weeks. We hypothesized that adolescents with full-syndrome BPD would self-report higher levels of depression at baseline, and that they would have less reduction in depressive symptoms over the trial period, compared to adolescents with sub-threshold BPD.

## Methods

### Participants and procedures

This study used data from a randomised controlled trial of a total of 77 adolescents, aged 12 to 18 years, on the efficacy of Dialectical Behaviour Therapy adapted for Adolescents (DBT-A) on the frequency of subsequent self-harm episodes, the level of suicidal ideation, and severity of depressive symptoms. For the purpose of this study only adolescents with a baseline diagnosis of depressive disorder (*N* = 39) were included. The depressive disorders comprised Major Depressive Disorder, Dysthymic Disorder, and Depressive Disorder Not Otherwise Specified. The adolescents were recruited from child and adolescent psychiatric outpatient clinics in the Oslo area. Inclusion criteria were a history of at least two episodes of self-harm, with at least one from the last 16 weeks; and at least two criteria of DSM-IV BPD (plus the self-harm criterion), or, alternatively, at least one criterion of DSM IV BPD plus at least two subthreshold-level criteria. A diagnosis of BPD required 5 or more BPD-criteria in addition to the general criteria for a personality disorder. The adolescents received either DBT-A or enhanced usual care (EUC) for 19 weeks, delivered by therapists working at, and funded by, ten child and adolescent psychiatric outpatient clinics participating in the study. The study sample and methods are described in detail elsewhere [27].

### Assessments

Assessments by interview and self-reporting were made by independent interviewers before randomisation, and 19 weeks after the first treatment session. Two child and adolescent psychiatrists and 2 doctoral-level clinicians conducted the baseline interviews. Two child and adolescent psychiatrists, one psychiatrist, and seven undergraduates from the Faculty of Medicine at University of Oslo, trained in relevant assessment-instruments and blinded to treatment allocation and to results from baseline interviews, conducted the interviews at trial completion. The Schedule for Affective Disorders-Present and Lifetime version (K-SADS-PL) [28] was used to obtain socio-demographic data, history of previous psychiatric treatment and DSM-IV axis I diagnoses, and the Structured Clinical Interview for DSM-IV (SCID-II) [29] was used to diagnose BPD. The Children’s Global Assessment Scale (C-GAS, range 0–100) [30] measured global level of functioning. The Lifetime Parasuicide Count (LPC) interview [31] was used to obtain history of self-harm. Severity of suicidal ideation was measured by the 15-item self-report Suicidal Ideation Questionnaire (SIQ-jr., range 0–90) [32]. Self-reported depressive symptoms were measured by the short (13-items) version of the Mood and Feelings Questionnaire (SMFQ, range 0–26) [33], and clinician-rated symptoms through the 10-item Montgomery Åsberg Depression Rating Scale (MADRS, range 0–60) [34]. All interviews were audio-taped, and inter-rater reliability (IRR) of diagnoses and outcome variables was checked by a child and adolescent psychiatrist expert in the relevant assessment instruments. Based on 26 IRR-rated interviews, mean Kappa was found to be 0.68 (range 0.50–0.81, *SD* = 0.10) for all symptoms rated with K-SADS-PL. Intra-class correlation (ICC) was used to test IRR for C-GAS (ICC = 0.42), MADRS score (ICC = 0.76), LPC (IRR = 0.99), and diagnostic criteria for BPD (ICC = 0.66). The internal consistency coefficients (Cronbach’s alpha) for the total MADRS score and the total SMFQ score were .79 and .81 respectively.

### Statistical analyses

Means and standard deviations are given for normally distributed variables. Medians and interquartile ranges are presented for non-normally distributed variables (lifetime numbers of self-harm episodes, number of Axis I disorders and number of BPD-criteria). Differences between the groups were tested using independent-sample t-tests and Mann-Whitney U-tests. Numbers and percentages are given for categorical variables. Differences between the group proportions were tested by Fisher exact tests. Changes in levels of depressive symptoms from baseline to trial completion were tested using paired samples t-tests. The total number of missing values was small. At baseline altogether four scores were missing of SIQ-jr. At trial completion, there were missing scores for two items of MADRS, two items of SMFQ, and three items of SIQ-jr. Since the sample size was limited and further analyses would be performed on the level of sum scores for SMFQ, MADRS and SIQ, the Expectation-Maximization (EM) algorithm with normal distribution was applied to impute the missing data instead of the multiple imputation method. Variables with *p*-value less than .05 were selected for inclusion in the multivariate linear regression. A series of forward stepwise multivariate linear regression was performed to examine the predictive ability of the selected variables. Regression coefficients with 95% confidence intervals, corresponding *p*-values and R-square as a measure for the predictive ability are given for three linear regression models. All tests were two-sided, and the significance level was set to .05. Analyses were performed with IBM Statistics 20.0 for Windows [35].

## Results

### Sample characteristics

The majority of the study participants were girls, and the mean age was almost 16 years (Table 1). All the adolescents with BPD were female, whereas 5 (17%) of the adolescents without BPD were male. On average, the individuals with BPD were significantly older than those without BPD, age range 14.6–18.9 vs 12.6–18.3. At baseline, participants had an average MADRS score of 22.1 (*SD* = 6.2) and an average SMFQ score of 16.3 (*SD* = 5.3), and these variables were not significantly correlated. Altogether 10 adolescents (26%) were diagnosed with BPD. The median number of DSM Axis I disorders at baseline was 2 (interquartile range = 2), with anxiety disorders as the most frequent co-morbid disorder (*n* = 19). Other co-morbid diagnoses were Post Traumatic Stress Disorder (PTSD) (*n* = 7), any eating disorder (*n* = 4), any substance abuse (n = 1), Attention-Deficit/Hyperactivity Disorder (ADHD) (*n* = 2), and Conduct Disorder (n = 2). The median number of self-reported life-time self-harm episodes was 49, with a wide interquartile range (98.5). The average baseline severity of suicidal ideation (SIQ-jr. = 39.9) was well above the clinical cut-off (score above 31) [32].

**Table 1:** Characteristics of the total sample of self-harming adolescents with depression, and a comparison between the adolescents with full-syndrome Borderline Personality Disorder (BPD) and with sub-threshold BPD at baseline and at trial completion

	Total		Full-syndrome BPD		Sub-threshold BPD		*P*
	n (%)		n (%)		n (%)		
Girls	34 (87.2)		10 (100)		24 (82.8)		.30
Receiving DBT-A treatment	21 (53.8)		5 (50.0)		16 (55.2)		1.0
History of psychiatric treatment	22 (57.9)		7 (70.0)		15 (53.6)		.47
5 BPD criteria or more	14 (35.9)		10 (100)		4 (13.8)		<.001
MDD	15 (38.5)		4 (40.0)		11 (37.9)		1.0
Dysthymia	6 (15.4)		1 (10.0)		5 (17.2)		1.0
Depression NOS	18 (46.2)		5 (50.0)		13 (44.8)		1.0
Any anxiety disorder	19 (48.7)		3 (30.0)		16 (55.2)		.27
	Mean (SD)	n_1_ + n_2_	Mean (SD)	n_1_	Mean (SD)	n_2_	
Age, years	15.8 (1.7)	39	16.9 (1.7)	10	15.5 (1.5)	29	.02
SMFQ							
Baseline	16.3 (5.3)	39	19.4 (4.7)^a^	10	15.2 (5.1)^c^	29	.03
-DBT		21	17.2 (3.0)	5	14.0 (5.4)	16	
-EUC		18	21.6 (5.4)	5	16.8 (4.5)	13	
Trial completion	12.3 (6.2)	39	16.5 (5.7)^a^	10	10.8 (5.8)^c^	29	.01
-DBT		21	15.2 (5.7)	5	9.1 (4.6)	16	
-EUC		18	17.8 (6.1)	5	12.9 (6.5)	13	
MADRS							
Baseline	22.1 (6.2)	39	22.2 (5.7)^a^	10	22.0 (6.4)^c^	29	.94
-DBT		21	26.2 (2.6)	5	21.6 (7.2)	16	
-EUC		18	18.2 (5.1)	5	22.6 (5.4)	13	
Trial completion	15.1 (8.1)	39	20.5 (6.3)^a^	10	13.3 (7.8)^c^	29	.01
-DBT		21	19.6 (8.6)	5	10.3 (7.2)	16	
-EUC		18	21.4 (3.8)	5	16.8 (7.2)	13	
C-GAS							
Baseline	53.3 (7.0)	39	52.1 (6.9)^b^	10	53.7 (7.1)^c^	29	.56
-DBT		21	52.6 (7.2)	5	54.0 (8.4)	16	
-EUC		18	51.7 (7.4)	5	53.2 (5.6)	13	
Trial completion	64.1 (11.5)	39	56.8 (6.6)^b^	10	66.6 (11.9)^c^	29	.003
-DBT		21	59.4 (6.6)	5	69.6 (9.2)	16	
-EUC		18	54.2 (6.1)	5	62.9 (14.0)	13	
SIQ-jr							
Baseline	39.9 (21.5)	39	53.5 (23.1)^b^	10	35.2 (19.1)^c^	29	.02
-DBT		21	45.3 (17.1)	5	30.3 (14.6)	16	
-EUC		18	61.8 (27.1)	5	41.2 (22.7)	13	
Trial completion	27.4 (20.0)	39	34.1 (21.3)^b^	10	25.1 (19.4)^c^	29	.23
-DBT		21	26.5 (6.3)	5	16.4 (11.6)	16	
-EUC		18	41.7 (28.9)	5	35.8 (21.9)	13	
Lifetime episodes of self-harm (n) ^d^	49.0, 98.5	39	89.0, 239.5	10	44.0, 76.3	29	.20
n. of Axis 1 Disorders^d^	2.0, 2.0	39	2.0, 2.0	10	2.0, 2.0	29	.89
n. of BPD-criteria^d^	4.0, 2.0	39	6.0, 2.0	10	3.0, 2.0.	29	<.001

^a^ A paired samples test showed a non-significant reduction in SMFQ (*p* = .16) and MADRS (*p* = .55) from baseline to trial completion

^b^ A paired samples test showed a significant reduction in SIQ-jr (*p* = .01) and CGAS (*p* = .04) from baseline to trial completion

^c^ A paired samples test showed a significant reduction in SMFQ (*p* = .001), MADRS (*p* < .001), CGAS (p < .001) and SIQ-jr (*p* = .04) from baseline to trial completion

^d^ median, interquartile range

### Diagnoses, depressive symptoms, and borderline criteria at baseline

In further analyses, the participants were divided into two groups; adolescents with full-syndrome BPD (*n* = 10) were compared with adolescents with sub-threshold BPD (*n* = 29). Self-reported and clinician-rated depressive symptoms were significantly and positively correlated in adolescents with sub-threshold BPD (Pearson’s *r* = .381, *p* = .04), but negatively inter-correlated in adolescents with full-syndrome BPD (Pearson’s *r* = −.466, *p* = .18), although this association was not statistically significant. There were no significant differences between the groups with regard to C-GAS, history of previous psychiatric treatment, or number or types of additional DSM IV diagnoses. The two groups did not differ with regard to baseline levels of clinician-rated depressive symptoms. However, adolescents with full-syndrome BPD had significantly higher levels of self-reported depressive symptoms and suicidal ideation compared to adolescents with sub-threshold BPD.

### Depressive symptoms at trial completion

Similar to the situation at baseline, self-reported and clinician-rated depressive symptoms at trial completion were significantly correlated only in adolescents with sub-threshold BPD (Pearson’s *r* = .638*, p < .001* vs Pearson’s *r =* .060, *p* = .87).

There were statistically significant between-group differences in levels of self-reported as well as clinician-rated depressive symptoms at trial completion (Table 1). The levels of both self-reported and clinician-rated depressive symptoms were significantly reduced from baseline to trial completion in adolescents with sub-threshold BPD, whereas none of the measures of depressive symptoms showed significant reductions in adolescents with full-syndrome BPD. A non-significant trend showed that adolescents with full-syndrome BPD who had received DBT-A (*n* = 5), had a 25% reduction in their clinician-rated depressive symptom scores over the trial period (mean = 26.2, SD = 2.6, to mean = 19.6, SD = 8.6,, d_Cohen_ = 1.3), whereas participants who had received EUC (n = 5) showed an 18% increase in this symptom level (mean = 18.2, SD = 5.1 to mean = 21.4, SD = 3.8, d_Cohen_ = 1.5) (Table 1). All the adolescents showed a significant reduction in levels of suicidal ideation; with no between-group difference. At trial completion the global level of functioning (C-GAS) was significantly lower in adolescents with BPD compared to those with sub-threshold BPD.

Baseline levels of clinician-rated depressive symptoms (MADRS), a diagnosis of BPD, and treatment condition (receiving DBT-A) were all univariately associated with levels of clinician-rated depressive symptoms at trial completion. These independent variables were all entered into a multivariate linear regression analysis with clinician-rated level of depressive symptoms at trial completion as the dependent variable (Table 2). A baseline diagnosis of BPD and high baseline levels of clinician-rated depressive symptoms predicted a poorer level of depressive symptoms at trial completion, whereas receiving DBT-A predicted a more favourable depressive symptoms score. Total R square adjusted for this model was 35.4, which is the percentage of total variation in MADRS-score at trial completion (Adjusted R square). Age was not a significant contributor to the outcome measure, and adding age in the final analysis did not change the explanatory power of the model (Adjusted R square = 35.1). As there were no boys diagnosed with BPD, a separate linear regression analysis including girls only was conducted, showing essentially the same result (data not shown).

**Table 2:** Association between Borderline Personality Disorder (BPD), Baseline Depressive symptoms (MADRS) and Treatment condition and Depressive Symptoms at trial completion^a^

	Step 1			Step 2			Step 3		
	β^b^	β (95% CI)	*p*	β^b^	β (95% CI)	*p*	β^b^	β (95% CI)	*p*
BPD	.40	7.26 (1.69–12.80)	.012	.38	6.99 (1.71–12.27)	.011	.38	6.87 (2.05–11.69)	.006
DBT-A				−.33	−5.31 (−9.94 --.69)	.025	−.37	−5.95 (−10.19- -1.71)	.007
MADRS baseline							.38	0.49 (.15–.84)	.007
Adjusted R square	13.6%			26.9%			35.8%		

^a^ Forward Multivariate Linear Regression Analyses of the Effect of a Baseline diagnosis of BPD, Treatment Condition and Level of Clinician-rated Depression on the level of Clinician-rated Depression (MADRS) at trial completion

^b^ Standardized regression coefficient

## Discussion

The three main findings of this study were that a) depressed adolescents with full-syndrome BPD self-reported significantly higher levels of depressive symptoms and suicidal ideation compared to adolescents with sub-threshold BPD whereas the two groups were rated as equally depressed by the clinicians, b) adolescents with full-syndrome BPD had a poorer treatment outcome in terms of higher levels of depressive symptoms and lower levels of global functioning at trial completion, and c) receiving DBT-A compared to EUC was associated with a greater reduction in depressive symptoms.

Previous studies on individuals with depression have reported discrepancies between self-reported and clinician-rating levels of depressive symptoms [36, 37], and that adults with co-morbid BPD rate their depressive symptoms as more severe compared to their clinicians’ ratings [24, 25, 38, 39]. Social desirability and limitations in self-observation skills have been found to influence self-report assessments in adults, and could have the same effect in adolescents although this has been less studied [22]. There are several possible explanations to the discrepancy between self-reported and clinician-rated depressive symptoms found in our study. First, according to Linehan [40], BPD is primarily a disorder of emotion regulation constituted by high emotional sensitivity, especially to negative emotional stimuli, more intense and more frequent responses to emotional stimuli, and a slow return to the emotional baseline. Individuals with BPD more often feel overwhelmed by their emotions, and their subjective experience of depression may thus be experienced as more intense or severe [24, 25, 41, 42]. Furthermore, as individuals with BPD typically have rapidly fluctuating emotions, self-reports may be more dependent on their present emotions, whereas the clinicians rate the severity of the patients’ depressive symptoms according to a longer time-frame. Another important contributor to the discrepancy between self-report and clinician rated levels of depressive symptoms may be the way individuals with BPD have been shaped by their environment to communicate about their symptoms and problems. According to the biosocial theory, BPD develops in a transaction between a child’s genetic vulnerability and an invalidating environment [40], e.g. that expressions of emotions are rejected by the family and life’s problems are oversimplified. Consequently, the child does not learn how to label and understand his or her emotional experiences, and is not taught how to modulate emotional arousal or cope with distress. In an invalidating environment, the child often learns that extreme emotional responses are needed to generate helpful responses. Thus adolescents with BPD may have communicated less effectively about their depressive symptoms in the interpersonal context of the interview in our study. Furthermore, since studies have shown that when individuals are perceived as dramatic, demanding or exaggerating, clinicians tend to ignore or underestimate the severity of their depressive symptoms [20, 43], we may speculate that clinicians of our study could have reacted in the same way. An additional possible explanation for the observed discrepancy between self-reported and clinician-rated depressive symptoms is that depressive symptoms in individuals with BPD are qualitatively different from the depressive symptoms in individuals with depressive disorders only. Depressive symptoms in individuals with in BPD may be more linked to feelings of emptiness and abandonment, negative emotions such as anger and hostility, and to hypersensitivity to inter-personal dilemmas [25, 44]; qualities that may be more difficult to rapidly capture by the clinicians. Finally, since the clinical presentations of depressive symptoms and BPD features are partially overlapping, clinicians as well as patients may have difficulties with distinguishing features of BPD from actual depressive symptoms [45].

In our study, adolescents with full-syndrome BPD had a significantly smaller reduction in depressive symptoms and less improvement in global functioning than adolescents without BPD. Our findings are in line with a recent study on depressive adolescents treated in a naturalistic outpatient setting [46], as well as with previous studies on adults [47–49]. Among possible explanations to this is that the presence of BPD is associated with poorer treatment-outcome than depression alone, and that remission of depression is predicted by improvements in BPD [8, 50, 51]. It is important to realize that 19 weeks of treatment could be too short a period to achieve significant improvements in BPD-pathology, and we would thus expect that depressive symptoms will remit more rapidly in individuals with sub-threshold BPD compared to individuals with full-syndrome BPD. An additional explanation may be that if we assume that adolescents with full-syndrome BPD tend to overestimate their level of depressive symptoms, they may also tend to underestimate their improvement in depressive symptoms. On the other hand, if we assume that adolescents with full-syndrome BPD may be more depressed than rated by the clinicians, their improvement may have been even poorer. Nevertheless, our findings suggest that receiving DBT-A is associated with lower levels of depressive symptoms at trial completion in both these groups. This could mean that providing BPD-specific treatment is important to achieve sufficient treatment response with regard to depressive symptoms in adolescents with BPD-traits, although firm conclusions cannot be drawn given the limited sample size. Although we still have sparse knowledge as to who may benefit most from which specialized treatment, there seems to be considerable agreement that the more severe and complex the condition the more specialized the treatment offered should be [52–55].

Our study does not offer any empirically founded answers to whether the discrepancies between self-reported and clinician-rated levels of depressive symptoms in adolescents with full-syndrome BPD come from the shortcomings of assessment tools, overrating by the adolescents, the failure of clinicians to capture participants’ level of depressive symptoms accurately, or whether there exist significant qualitative differences in depressive symptoms in adolescents with and without BPD. Both self-report and clinician-rated scales have their methodological limitations. Although it has been suggested that clinician-rated scales should be used as the principal outcome measure in clinical settings, self-reports contribute valuable information and thus may provide a complementary view [20]. More research, including larger samples of adolescents, is needed, and should include studies on specific depressive symptoms. Furthermore, new methodology, such as Ecological Momentary Assessment [56], which involves repeated sampling of subjects’ current behaviors and experiences in real time in their natural environments, could be a useful approach to detangle the different aspects of depression and emotion regulation and their temporal and possibly casual associations.

## Limitations and strengths

The limited sample size of this study warrants caution in the interpretation of results. The small sample size could have increased the probability of Type II errors, thus important associations may be undetected. Furthermore, the true differences between the sub-threshold BPD-group and the full-syndrome BPD-group and the true value of receiving DBT-treatment could not be identified due to the wide confidence intervals. The study was conducted within the context of a specialized treatment trial, with a predominantly female sample, and all of the adolescents had depressive disorders and BPD-traits. Thus, despite the fact that inclusion criteria were fairly wide, results cannot be directly generalized to other treatment settings or to self-harming adolescents in general and one must be careful about generalizations to male adolescents. Diagnostic validity and clinical utility of existing cut-offs for a diagnosis of BPD have been questioned in adolescents [57]. However, our findings suggest that there are important differences even between the adolescents with sub-threshold BPD and those with full-syndrome BPD. One may speculate whether the differences between the groups would have been larger if the adolescents with full-syndrome BPD were compared with adolescents without features of BPD, but with depression only. Among the strengths of the study are the absence of dropout from follow-up, the application of rigorous procedures for data collection, the integrity of ratings, and blinding and independence of raters.

## Conclusion and clinical implications

In self-harming adolescents with depressive disorders the presence of full-syndrome BPD seems to have significant implications for both self-reported and clinician-rated assessment of depressive symptoms, as well as for the treatment response. Clinicians should therefore adopt rigorous assessment methods for evaluation of depressive symptoms and be alert to the possibility of BPD in self-harming adolescents. This study suggests that using both self-report and clinician-ratings in assessing symptoms of depression are of considerable clinical value since these dual measurements may capture different aspects of depressive symptoms. Significant discrepancies between self-reported and clinician-rated levels of depressive symptoms in self-harming adolescents are important to detect and should lead to a closer evaluation as they may be suggestive of personality problems. Furthermore, clinicians should be aware of the possibility of underestimating the severity of depression in the context of emotional and behavioral dysregulation. Finally, exploring such discrepancies explicitly with the adolescents in the therapeutic work may help the clinician to better understand and treat adolescents’ depressive symptoms.

## Abbreviations

BPD: Borderline Personality Disorder
C-GAS: The Children’s Global Assessment Scale
DBT: Dialectical behavior therapy
Depression NOS: Depressive Disorder Not Otherwise Specified
DSM- IV: Diagnostic and statistical manual of mental disorders fourth Edition (Text revision)
EUC: Enhanced usual care
IRR: Inter-rater reliability
K-SADS-PL: The Schedule for Affective Disorders-Present and Lifetime version
LPC: The Lifetime Parasuicide Count
MADRS: Montgomery Åsberg Depression Rating Scale
MDD: Major Depressive Disorder
RCT: Randomized controlled trial
SCID-II: Structured clinical interview for DSM-IV axis II disorders
SIQ-jr: Suicidal Ideation Questionnaire
SMFQ: Short mood and feelings questionnaire
